# A167 HISTOLOGICAL REMISSION PLACEBO RATES IN ULCERATIVE COLITIS TRIALS: A SYSTEMATIC REVIEW AND META-ANALYSIS

**DOI:** 10.1093/jcag/gwac036.167

**Published:** 2023-03-07

**Authors:** M Youssef, K Dong, S J Lee, N Narula

**Affiliations:** 1 Internal Medicine, University of Toronto, Toronto; 2 General Surgery; 3 Gastroenterology, McMaster University, Hamilton, Canada

## Abstract

**Background:**

High histologic remission rates have been reported with placebos in randomized controlled trials (RCTs) evaluating ulcerative colitis (UC) therapies and have varied based on trial designs. We performed a systematic review and meta-analysis to quantify placebo histological remission rates and identify factors influencing those rates.

**Purpose:**

This systematic review aims to improve future trials design and minimize placebo rates in UC trials.

**Method:**

MEDLINE, EMBASE, and the Cochrane library were searched from inception of the databases until December 2021. We included placebo-controlled RCTs of adult patients with UC treated with aminosalicylates, corticosteroids, immunosuppressives, biologics, and small molecules. We pooled estimates using a random-effects model and performed subgroup analysis as well as meta-regression to evaluate the effect of different covariates on placebo rates.

**Result(s):**

Thirty-three studies (30 induction and 3 maintenance) were included. The overall placebo histological remission rate was 15.7% [95% CI 12.9-19%] across all 33 studies (Figure). High heterogeneity was observed among studies with I^2^ = 62.10%. In induction studies, the pooled estimate of histological remission was 15.8% [95% CI 12.7-19.5%], while in maintenance studies the pooled estimate was 14.5% [95% CI 8.4-24%]. Subgroup analysis revealed statistically significant differences in placebo rates when accounting for background medications, the intervention drug class, and disease severity [p= 0.041, 0.025, and 0.025, respectively]. There was no statistical difference between induction vs. maintenance studies or between different histological scales [p= 0.771, and 0.075, respectively]. Meta-regression showed similar results except that the therapy used was not statistically significant [p-value= 0.059].

**Image:**

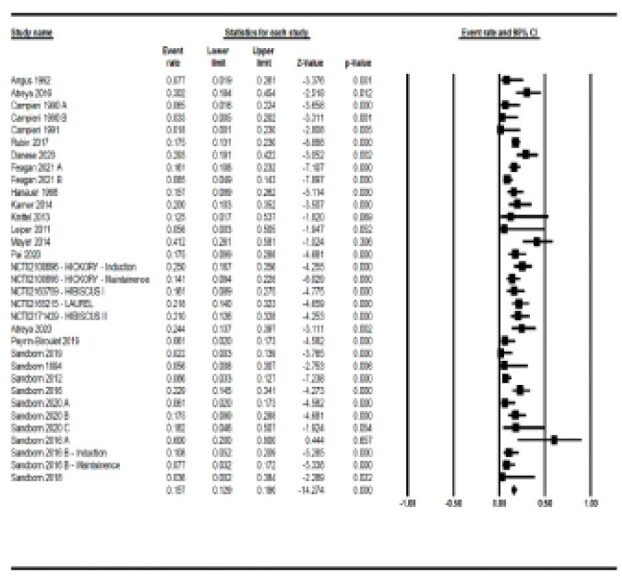

**Conclusion(s):**

Placebo histological remission rates range from 13-19% in UC RCTs, but studies are highly heterogeneous. Factors found to influence placebo rates include presence of background medications, the drug used and the disease severity in UC patients. These observations have important implications in informing future trial designs to minimize placebo rates and reduce heterogeneity.

**Disclosure of Interest:**

M. Youssef: None Declared, K. Dong: None Declared, S. J. Lee: None Declared, N. Narula Speakers bureau of: received honoraria from Janssen, Abbvie, Takeda, Pfizer, Merck, Sandoz, Novartis, and Ferring

